# A New Risk-Scoring System for Colorectal Cancer and Polyp Screening

**DOI:** 10.5152/tjg.2022.22431

**Published:** 2022-12-01

**Authors:** Alexandra Romanová, Jan Šťovíček, Jan Brož

**Affiliations:** 1 Department of Internal Medicine, Second Faculty of Medicine, Charles University, Prague, Czech Republic

Dear editor,

We read with interest the article “A New Risk-Scoring System for Colorectal Cancer and Polyp Screening by Turkish Colorectal Cancer and Polyp Study Group” by Erdem et al^1^ published online in this journal. The article presents an analysis of several risk factors of colorectal cancer and the results of colonoscopy of 6508 subjects suggesting a new scoring system for colorectal cancer and polyp screening. The authors concluded that according to this new scoring system, screening for colorectal cancer should start at the age of 45 in patients with scores ≥4.

The authors should be congratulated on their efforts in conducting such an important study and analysis of a large amount of data and highlighting a very clinically relevant and prevalent topic.

Although we agree with many of the conclusions, we would like to make some comments.

The number of points assigned to body mass index, male sex, and smoking does not correspond between the text (methods) and Table 1. Also, it would be interesting to see the logistic regression analysis results with the odds ratios.

There is an ambiguous definition of overweight status which should be explained.

Should also be mentioned among the study limitations that the study participants were preselected by general practitioners, and thus, the study results may not entirely correspond with a whole population data.

Available data show that patients with diabetes have an increased risk of colorectal adenoma and carcinoma and an increased risk of colorectal carcinoma at a lower age.^2-4^ We wonder if the data related to the diabetes history are available as these might further increase the study impact.

We respectfully suggest taking these points into account if the continuation of this important study is planned.

## Author’s Reply

**Re: A New Risk-Scoring System for Colorectal Cancer and Polyp Screening**

To the editor,

We appreciated Romanova et al^1^ for their comments regarding our study titled “A New Risk-Scoring System for Colorectal Cancer and Polyp Screening by Turkish Colorectal Cancer and Polyp Study Group”.^2^ We offer the following in reply.

In material method section we explained that based on the TCS scoring system; the age, gender, family history of colon cancer, body mass index (BMI), smoking status, and alcohol status were included in this analysis. However, there is no alcohol use among the parameters in Table 1 showing the scoring system. In this study we also questioned the amount of alcohol use by the patients. However, after the data were collected, this parameter was not included in the scoring system because there was a lack of information on alcohol use. Nevertheless, this deficiency does not affect our results, as the scoring system is a newly created system based on our data. In addition, in this study our primary goal was to develop a simple and usable scoring system. Therefore, we did not want to do too much statistical analysis. However, we will take your suggestion into consideration in our ongoing studies.

The authors are right about the emphasis on “ambiguous definition” of obesity. But in our simple and useful scoring system BMI over 30 is one point, below 30 is zero point. As a result, this methodology does not affect our results. Our main goal here was to distinguish between obese and non-obese patients more clearly and accurately while evaluating the demographics data.

In this study the patients were selected by gastroenterology specialist therefore our results reflect the whole population data.

To date most of the studies have shown a link between diabetes mellitus, hyperglycemia, and colorectal cancer.^3,4^ Therefore, recording the presence of diabetes mellitus in the data analysis of the patients will help us to obtain very important results. This suggestion is a very valuable and appropriate advice and will definitely be evaluated in further studies.

Levent Erdem, Erdem Koçak

Department of Gastroenterology, Demiroğlu Bilim University Faculty of Medicine, İstanbul, Turkey
